# Unusual axillary metastasis of recurrent nasopharyngeal cancer: A case report

**DOI:** 10.1097/MD.0000000000006854

**Published:** 2017-05-19

**Authors:** Deng-Yu Kuo, Min-Hsiang Chang, Shan-Ying Wang, Pei-Ying Hsieh, Pei-Wei Shueng

**Affiliations:** a Division of Radiation Oncology; b Department of Anatomical Pathology; c Division of Nuclear Medicine, Far Eastern Memorial Hospital, New Taipei City; d Department of Biomedical Imaging and Radiological Sciences, National Yang-Ming University, Taipei; e Division of Medical Oncology, Far Eastern Memorial Hospital, New Taipei City; f Faculty of Medicine, School of Medicine, National Yang-Ming University, Taipei; g Department of Radiation Oncology, Tri-Service General Hospital, National Defense Medical Center, Taipei, Taiwan.

**Keywords:** axillary metastases, chemoresistance, recurrent nasopharyngeal cancer

## Abstract

**Rationale:**

Nasopharyngeal carcinoma (NPC) has a high propensity of metastasis. The most commonly described sites of distant metastasis are the bones, lungs, and liver, whereas axillary metastasis is seldom reported.

**Patient concerns:**

We hereby present the case of a 66-year-old man with NPC, cT2N2M0, at diagnosis. He had completed chemoradiotherapy and been disease-free for 7 years.

**Diagnoses:**

After that period, late recurrence in the form of a solitary axillary lymph node metastasis was detected and confirmed by core-needle biopsy.

**Interventions:**

The lesion was chemoresistant but responded to salvage radiotherapy at a dose of 65 Gy in 21 fractions.

**Outcomes:**

Post-radiotherapy positron emission tomography scan showed no evidence of disease.

**Lessons:**

We suggested that long-term follow-up of NPC patients is important because a late relapse may occur at an unusual site. Aggressive management of solitary metastasis may achieve good outcome.

## 1 Introduction

Nasopharyngeal carcinoma (NPC) is a common head and neck cancer in Southeast Asia. It has a high propensity of metastasis, including an 87% chance of neck lymphadenopathy and a 3% to 6% chance of being accompanied by distant metastases at presentation.^[1]^ Although good locoregional control can be achieved by chemoradiotherapy (CRT), 18% to 50% of NPC patients will develop distant failure during the disease course. The most commonly described sites of distant metastasis are the bones, lungs, and liver. NPC with axillary nodal metastases has rarely been reported, and most of these cases had diffuse distant spreads or recurrent disease at the primary site.^[2–4]^ Herein, we present a case of solitary axillary lymph node recurrence in a NPC patient treated by CRT. Ethical approval for this study (105034-C) was provided by the Ethical Committee of Far Eastern Memorial Hospital on May 17, 2016. Written informed consent from the patient was obtained.

## 2 Case report

A 66-year-old man without any major systemic diseases initially presented with a mass on the left side of the neck in 2007. After biopsy and imaging studies, he was diagnosed with a T2N2M0, nonkeratinizing undifferentiated carcinoma of the nasopharynx. He completed a full course of definitive treatment that included induction chemotherapy (2 cycles of epirubicin, cisplatin, leucovorin, and 5-fluorouracil [5-FU]), followed by concurrent CRT (radiotherapy [RT] with 70.2 Gy in 39 fractions and 2 cycles of cisplatin, leucovorin, and 5-FU). 6-MV photons were used to treat the nasopharynx and regional lymphatics with intensity-modulated RT and sequential boost technique. A total dose of 70.2 Gy was delivered to the gross lesions and 45 to 61.2 Gy to the elective regions. The treatment plan was performed using the Pinnacle3 version 7.6c planning system (Philips Medical Systems, Andover, MA). After the treatment, both the primary tumor and regional lymph nodes showed a complete response and there was no evidence of disease until October 2014.

**Figure 1:**
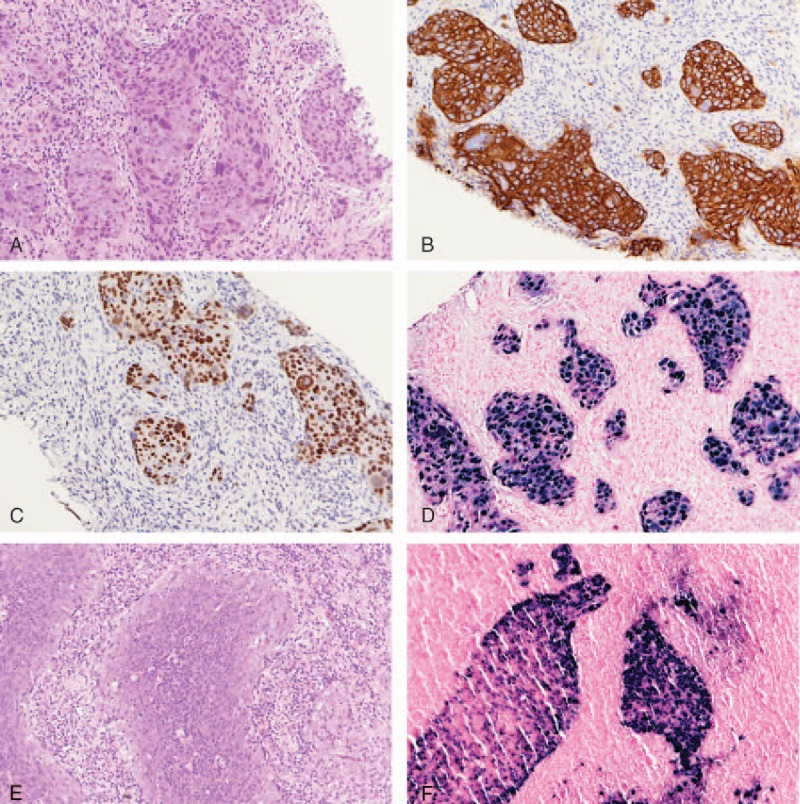
Pathohistochemical analyses of a core needle biopsy of the axillary metastasis. Microscopically, the epithelioid tumor cells were arranged in nests infiltrating into the desmoplastic stroma. They had an abundant cytoplasm and vesicular nuclei with prominent nucleoli (A). The neoplastic cells were immunoreactive to cytokeratin (B) and p63 (C), whereas the Epstein–Barr encoding region (EBER) in situ hybridization exhibited nuclear staining (D). The morphology and EBER in situ hybridization pattern were identical to the NPC tumor specimen at the time of initial diagnosis (E, F). EBER = Epstein–Barr encoding region.

At this time, a palpable mass in the left axillary region was detected. Chest computed tomography showed a large lobulated 9-cm mass lesion; therefore, differential diagnoses of lymphadenopathy, lymphoma, and malignant fibrous histiocytoma were proposed. There were no obvious breast or lung lesions, and the NPC remained under good locoregional control. For further confirmation, echo-guided core needle biopsy was performed. Pathological examination of the biopsy revealed a metastatic poorly differentiated carcinoma, of probable NPC origin (Fig. 1).

After diagnosis of metastatic NPC, the patient received 2 cycles of chemotherapy consisting of epirubicin, cisplatin, leucovorin, and 5-FU. He exhibited symptoms of severe oral mucositis and deep vein thrombosis, and the tumor progressed during the course of treatment. Thus, chemotherapy was discontinued and RT was administered for the treatment of the solitary lesion in the left axilla. The RT was delivered with helical tomotherapy (Hi-Art system, TomoTherapy, Madison, WI), which had *high conformality and reduced toxicity.* After the patient received 50 Gy in 16 fractions, no change in tumor size was observed. The option of salvage surgery was offered to the patient, but he declined it after being informed that there was a high risk of treatment-related lymphedema or brachial plexus and vascular injury.

**Figure 2:**
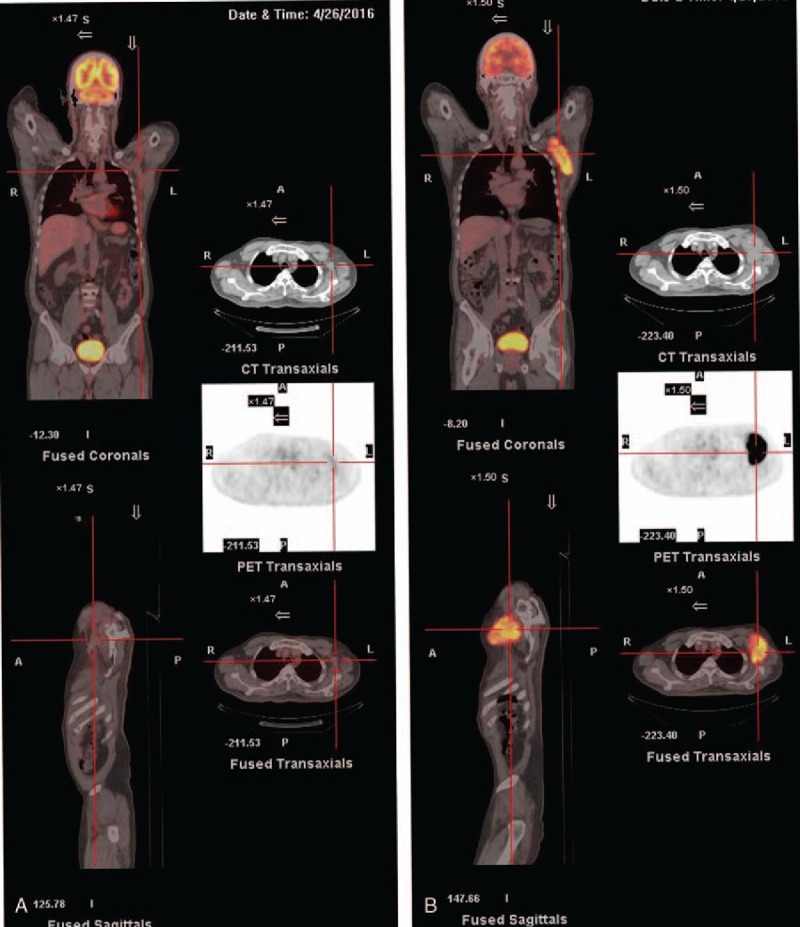
A comparison of the pre-treatment (A) and post-treatment (B) states by positron emission tomography (PET). Fluoro-deoxy glucose (FDG) studies marked a decrease in both the size and metabolism index of the left axillary soft tissue. The standardized uptake value (SUV) dropped from a pre-treatment value of 11.0 to a post-treatment value of 2.2. Minimal residual metabolism was detected in the field, which indicated a complete metabolic response. FDG = fluoro-deoxy glucose, PET = positron emission tomography, SUV = standardized uptake value.

Three months later, a gradual regression of the tumor was noted. A further boost of RT (15 Gy in 5 fractions) was administered, followed by positron emission tomography (PET). The PET scan showed no active lesions in the axilla or other visceral organs (Fig. 2). Currently, the patient has been disease-free for 17 months. He has not experienced radiation-induced arm lymphedema. Instead, he has grade 2 subcutaneous tissue fibrosis in the left axilla region without functional impairment.

**Figure 3:**
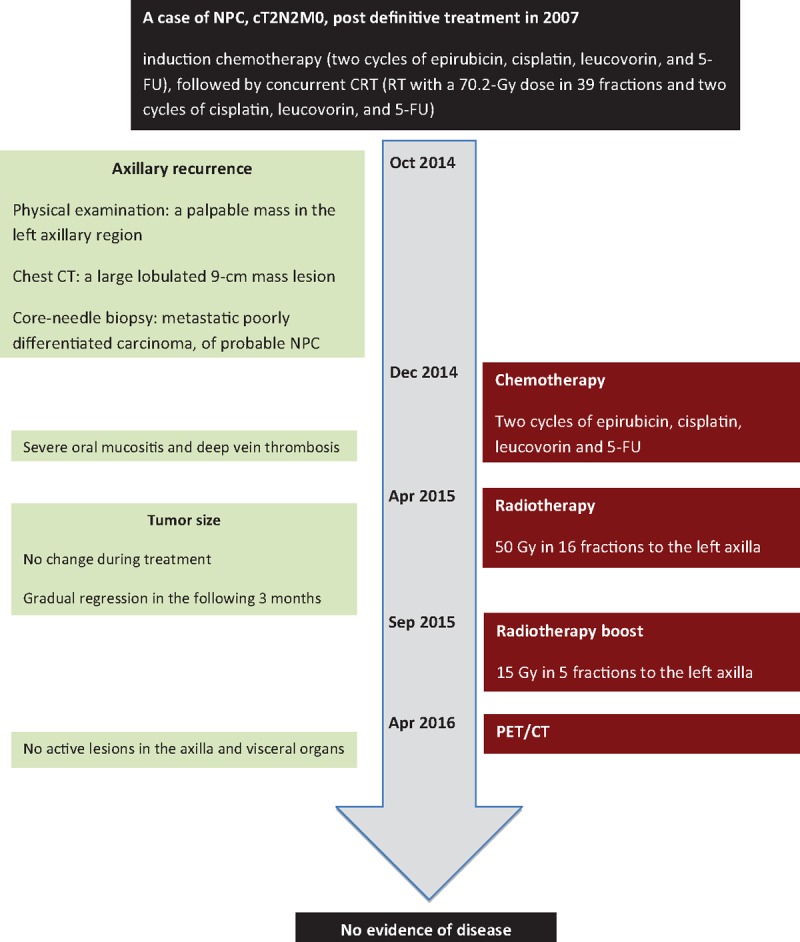
A timeline of this case.

A timeline of the important milestones related to the diagnoses and interventions is presented in Fig. 3.

## 3 Discussion

Non-mammary metastases to the breast and axilla rarely occur. The Memorial Sloan-Kettering Cancer Center searched its pathology database from 1990 to 2010 and identified only 85 cases of such metastases.^[5]^ Excluding hematological malignancies, the most common tumor types in the 85 cases were ovarian carcinoma (29%), lung carcinoma (22%), and melanoma (21%). Head and neck carcinoma accounted for 8% of the cases, but there was no case with an NPC origin.

The John's Hopkins University reported 4 cases of axillary metastases arising from head and neck cancer.^[2]^ In each case, the disease course was protracted, the neck lymphatics had been manipulated, and a recurrent or second primary mucosal lesion predated the axillary metastases. In our case, both sides of the neck had been treated with an RT dose of at least 45 Gy in the first course of CRT, as a prophylactic measure. The axillary lymph node metastasis occurred 7 years later, in accordance with a protracted disease. This was the only recurrence experienced by the patient; in other words, he had a solitary late axillary relapse from a primary NPC, which has not been previously reported in the literature.

NPC with a solitary metastasis is sensitive to aggressive and potentially curative treatment.^[6,7]^ Long-term survival is not uncommon in patients with this disease, and the prognosis is generally best in those with lung metastasis. Local treatment plus chemotherapy provides a survival benefit compared with chemotherapy alone, especially in patients with long disease-free intervals. As previous studies reported, metastatic NPC is most commonly treated via platinum-based chemotherapy, with a 50% to 90% overall response rate.^[8]^ For tumors showing drug resistance, especially those expressing multi-drug resistance gene 1, poor overall survival is predicted.^[9]^ In our case, the recurrent axillary tumor initially showed no response to chemotherapy, so the treatment strategy was modified to include salvage RT. A delayed tumor response was observed after 3 months of RT. Although NPC is very radiosensitive, the speed of tumor regression can vary. In a retrospective study, about 15% of primary tumors were in the form of a stable disease at the end of RT, but only 2.6% of patients had residual tumors 3 to 4 months after RT.^[10]^

For most oncologists, concurrent CRT has been accepted as the treatment of advanced NPC. Whether adding adjuvant chemotherapy provides further benefits is debatable. In a phase 3 trial of patients with locoregionally advanced NPC, adjuvant cisplatin and 5-FU chemotherapy after concurrent CRT did not significantly improve the 2-year failure-free survival rate.^[11]^ Nor did adjuvant chemotherapy contribute an overall survival in a recent meta-analysis of patients with non-metastatic NPC.^[12]^ However, several retrospective studies recommend adjuvant chemotherapy in addition to concurrent CRT in selected high-risk patients because distant metastasis is still the major failure pattern when using the modern radiation technique.^[13]^ Thus, the absence of adjuvant chemotherapy might be one of the reasons for the late recurrence in our case.

Recently, both PET and quantification of plasma Epstein–Barr virus (EBV) DNA have demonstrated encouraging efficacy in NPC staging, response evaluation, and relapse detection. In lesion-based analyses for detection of relapses, the sensitivity of PET and plasma EBV DNA quantification was 81.8% and 100%, respectively.^[14]^ Furthermore, elevated EBV DNA levels may a better indicator of NPC than is PET, with earlier detection and before the appearance of clinical symptoms. The guidelines of the National Comprehensive Cancer Network recommend using EBV DNA as a marker for NPC.^[15]^ In routine follow-ups at our hospital, these 2 tools were not used. Instead, PET was performed in order to confirm the absence of disease after salvage RT.

This report indicates that regular follow-up of NPC patients is important because late relapse may occur at an unusual site. Even 5 years after definitive treatment, once per year follow-ups are needed.^[15]^ Even if a tumor is resistant to chemotherapy, there is still a chance to salvage it by RT, and thus, the optimal strategy for each recurrent NPC case should be determined by a multidisciplinary team.

## Acknowledgements

The authors would like to thank Editage (www.editage.com) for English language editing.
